# The Clinical Implications of KRAS Mutations and Variant Allele Frequencies in Pancreatic Ductal Adenocarcinoma

**DOI:** 10.3390/jcm13072103

**Published:** 2024-04-04

**Authors:** Faria Nusrat, Akshay Khanna, Aditi Jain, Wei Jiang, Harish Lavu, Charles J. Yeo, Wilbur Bowne, Avinoam Nevler

**Affiliations:** 1 Sidney Kimmel Medical College, Thomas Jefferson University, Philadelphia, PA 19107, USA; faria.nusrat@students.jefferson.edu (F.N.); charles.yeo@jefferson.edu (C.J.Y.);; 2 Jefferson Pancreas, Biliary and Related Cancer Center, Department of Surgery, Thomas Jefferson University, Philadelphia, PA 19107, USA; 3 Sidney Kimmel Cancer Center, Department of Pathology, Thomas Jefferson University, Philadelphia, PA 19107, USA

**Keywords:** pancreatic cancer, oncologic outcomes, KRAS, mutant subtype, variant allele frequency

## Abstract

The KRAS proto-oncogene is a major driver of pancreatic tumorigenesis and is nearly ubiquitously mutated in pancreatic ductal adenocarcinoma (PDAC). KRAS point mutations are detected in over 90% of PDAC cases, and these mutations have been shown to be associated with worse therapy response and overall survival. Pathogenic KRAS mutations are mostly limited to codons 12, 13 and 61, with G12D, G12V, G12R, Q61H, and G13D accounting for approximately 95% of the mutant cases. Emerging data have shown the importance of specific mutant subtypes, as well as KRAS variant allele frequency on clinical prognosis. Furthermore, novel technologies and therapies are being developed to target specific mutant subtypes, with encouraging early results. In this paper, we aim to review the recent studies regarding the relative impact of specific mutant KRAS subtypes on oncologic outcomes, the application of variant allele frequency in next generation sequencing analyses, and the ongoing research into therapies targeting specific mutant KRAS subtypes.

## 1. Introduction

Pancreatic cancer is an aggressive malignancy with an overall 5-year survival rate that has only recently improved to 13%. Even early stage pancreatic cancer that is amenable to surgical resection and chemotherapy is still associated with survival rates below 50% at 5 years (Table 1). While the overall 5-year survival rates have improved over the past decades to 13%, the overall incidence of pancreatic cancer has unfortunately more than doubled in the past 30 years [1]. Additionally, due to the insidious nature of this cancer, less than 20% of patients are potential candidates for resection, as most patients are diagnosed after the cancer has already spread regionally or distally.

**Figure 1:**
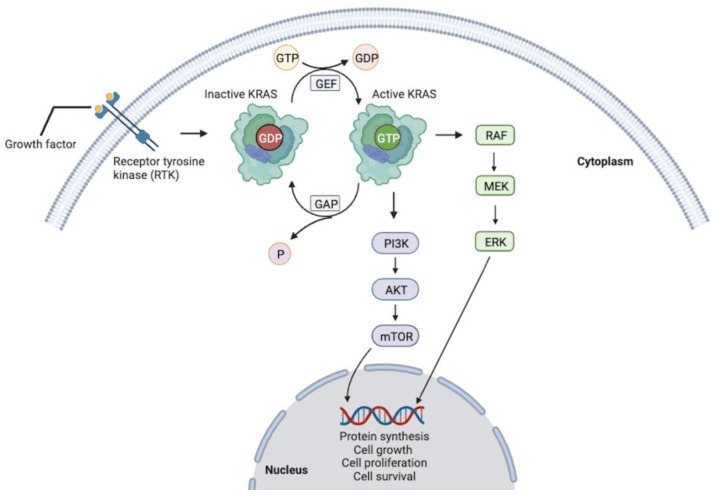
Mutated RAS drives cancer through a constitutively active GTP-bound state, promoting unregulated intracellular signaling.

Pancreatic ductal adenocarcinoma (PDAC) arises from the ductal epithelium of the exocrine pancreas and represents greater than 90% of all diagnosed pancreatic cancers [1]. Approximately 85 to 95% of PDAC cases have a KRAS (Kirsten rat sarcoma) proto-oncogene mutation, serving as an important driver of disease initiation and progression [2,3,4]. KRAS is a member of the RAS family of guanosine nucleotide-binding proteins that activate intracellular signaling pathways, notably the MAPK and mTOR pathways, regulating cellular growth, proliferation, differentiation, and survival [2,4]. RAS GTPases are normally found alternating between the active GTP- and inactive GDP-bound forms. Guanine nucleotide exchange factors (GEFs) activate the GTPase by facilitating the exchange of GDP for GTP. Conversely, GTPase-activating proteins (GAPs) catalyze GTP hydrolysis into GDP (Figure 1). However, mutations can lead to constitutive activation and thus unregulated signaling that is characteristic of cancer.

**Figure 2:**
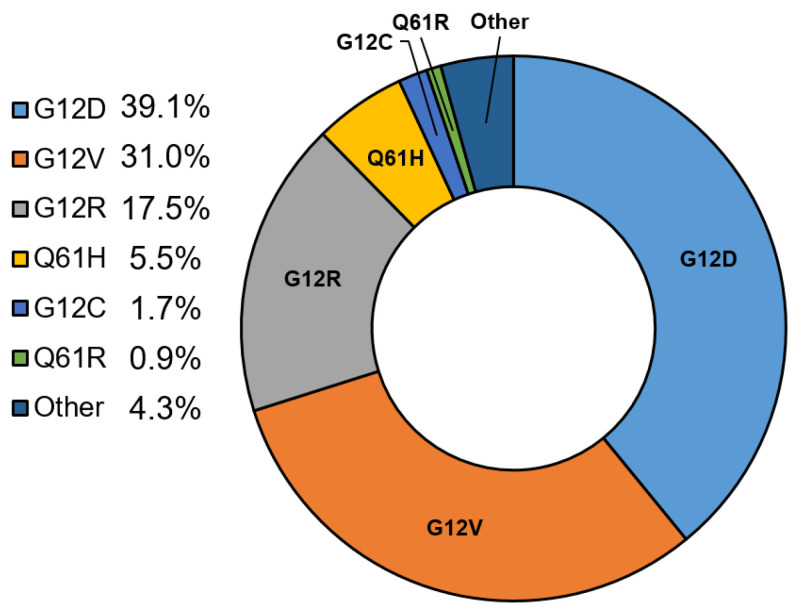
Distribution of mutant KRAS variants in clinical samples (*n* = 348) of pancreatic ductal adenocarcinoma (queried from www.cBioPortal.org QCMG data, 21 March 2024).

Missense mutations in the *KRAS* gene (mKRAS) most frequently occur at codons 12, 13, or 61 in about 95% of cases [2]. The most common tumor-associated KRAS mutations include G12D (39.2%), G12V (32.5%), G12R (17.1%), and Q61H (4.8%) [4]. Review of other publicly available databases, such as the QCMG [5] (available on www.cBioPortal.org, accessed on 21 March 2024), reveals similar distributions (Figure 2). These single amino acid substitutions in glycine-12 (G12) and glycine-13 (G13) decrease GAP binding, while glutamine-61 (Q61) mutations impair the intrinsic GTPase function of KRAS, with both mechanisms ultimately resulting in a stable GTP-bound KRAS and overactivation of the RAS pathway. Different mutations can change the intrinsic KRAS GTPase activity as well as result in variable binding affinity of the regulatory factors (GAPs/GEFs). This supports the rationale for variable disease phenotypes dependent on the specific mutant KRAS subtype [6]. Despite different protein loci of the various mutations, there are conflicting data on the impact of specific mutations on the oncologic outcomes in pancreatic cancer. This review will discuss the clinical impact of KRAS mutation status and the outlook of using KRAS mutation information to detect and treat pancreatic cancers.

## 2. Review Methodology

PubMed was queried using the following with the Mesh terms: “Pancreatic Neoplasm”, “Mutation”, “Prognosis”, and “KRAS”. To narrow the results relating to specific topics regarding the prognostic impact of KRAS variant allele frequencies and current clinical trials, we included additional Mesh terms to our previous search criteria in PubMed: “Gene Frequency” and “Allele Frequency” as well as “Clinical trial”. In total, 130 PubMed results were reviewed in preparation of this review.

## 3. Clinical Impact of KRAS Variants

With the increased adoption of advanced sequencing techniques, multiple studies have sought to understand the impact of the KRAS mutation subtype on oncologic outcomes, such as survival, recurrence, and metastasis. Mutant KRAS tumors greatly differ from KRAS wild-type tumors in their mutational landscape. Mutant KRAS tumors are highly associated with other classic driver mutations such as *TP53*, *SMAD4*, and *CDKN2A*, while KRAS wild-type tumors are characterized by downstream RAS/RAF/MAPK/PI3K-activating mutations, increased mutation rates of *ERBB2* (HER2) and *ATM*, and increased tumor mutational burden (TMB) [7,8]. 

KRAS wild-type (wtKRAS) pancreatic cancer patients have been consistently shown to have improved overall survival and disease-free survival [9,10,11,12,13,14,15]. For example, in patients with advanced, unresectable pancreatic cancer, wild-type KRAS status was associated with improved survival compared with mutant KRAS, as reported by Ogura et al. [13] (15.5 months vs. 8.4 months, *p* = 0.03). Similarly, McIntyre and colleagues have shown, in their study of PDAC patients with early stage, resectable PDAC, a significantly poorer survival of patients with mutant KRAS than patients with wild-type KRAS (38.8 months vs. 91.0 months, *p* = 0.043) [14]. In a different approach, Masetti et al. performed a comparison between groups of long-term survivors and short-term survivors, characterizing differences in the genetic profiles of the tumors. Their study has similarly found that long-term survivors were associated with lower rates of KRAS mutations [11]. However, several retrospective analyses have failed to show such a difference, possibly suggesting that the specific mutation subtype is more informative than the mere existence of a mutation [16,17,18]. Supporting this idea are studies assessing the relationship between KRAS mutant subtypes and histological phenotypes. Dai et al. noted that KRAS G12D mutations were associated with an increased rate of poorly differentiated tumors, as compared with wild-type KRAS [9]. Moreover, Yousef et al. noted that KRAS G12R was associated with an increased incidence of well to moderately differentiated tumors [15].

A study by Hendifar et al. [19] that assessed 1475 patients with advanced-stage PDAC revealed that the KRAS G12R and G12V mutations had the greatest overall survivorship, while it appeared that KRAS G12D portended the worst prognosis and could serve as an independent factor in both locally advanced and metastatic PDAC [9,17,19]. All KRAS mutation subtypes were found to have a worse prognosis than wild-type KRAS. In addition, no significant difference in overall and disease-free survival was found by Hendifar et al. between mutations on the KRAS 12th codon and mutations on the 61st codon when the mutations were analyzed by location rather than subtype [19]. Another study by Ogura et al. that assessed 242 patients with unresectable pancreatic cancer found that patients with the G12V mutation had a longer overall survival than patients with G12D or G12R but found no significant difference in the survivorship between patients with G12D and G12R [13]. These findings are corroborated by an earlier study of 157 patients who had undergone resection for PDAC where it was discovered that the G12V mutation had a longer median survival than those with the G12D or G12R subtype [20]. Recently, Yousef et al. [15] analyzed a large cohort from the MD Anderson Medical Cancer Center (*n* = 803) composed of stages I–IV pancreatic cancer patients. Both G12D and Q61H mutant subtypes were noted to correlate with significantly poorer survival as compared to wild-type KRAS. They proceeded to analyze the PanCAN “Know Your Tumor” (KYT) dataset (*n* = 408), finding the G12R subtype to have a survival advantage as compared with G12D and Q61H. Diehl et al. have also reported improved survival in PDAC patients with G12R tumors; however, it is important to note that their G12R cohort was also significantly enriched with tumors with homologous repair deficiency, which may confound the true impact of the subtype.

In an early study from the year 2000 assessing resectable PDAC patients, G12D was found to correlate with worse overall survival [20]. This observation has since been corroborated multiple times, with Shen et al. reporting similar findings in resectable pancreatic cancer patients (G12D: 11.7 months median survival vs. non-G12D: 26.6 months, *p* = 0.019) [21]. However, in a recent study, Shoucair et al. suggested that the interaction of G12D with other key driving mutations (such as TP53) is more impactful than KRAS subtype alone [22].

Several studies have assessed the utility of mutant KRAS subtyping in circulating tumor DNA (ctDNA). Guo et al. found that mutant KRAS, and especially the G12D subtype, corresponded with early metastasis and worse overall and recurrence-free survival in patients after curative-intent pancreatic resection [23]. Chen et al. assessed the impact of mutant KRAS detection in ctDNA in unresectable PDAC. KRAS mutations and specifically codon 12 mutations were strongly correlated with poor survival [12]. A summary of these studies is provided in Table 2.

## 4. Clinical Impact of KRAS Variant Allele Frequency

While it is accepted that the presence of a KRAS mutation confers a poor prognosis, Lennerz and Stenzinger [25] suggested that the mutant/variant allele frequency (VAF) has a far greater impact than the mere existence of the KRAS mutation. They have noted that improved next-generation sequencing techniques allow better detection of *KRAS* mutant alleles, thereby increasing the rate of KRAS mutations from 80% to 90%. Furthermore, their re-examination of the Biankin dataset [5] suggested that tumors with low mutant allele frequency, when corrected for tumor cellularity, are possibly associated with better survival outcomes (though they failed to reach statistical significance in their analysis). This also explains the discrepancy between earlier reports estimating *KRAS* mutation rates in PDAC at only 80 to 85% [24] compared with newer reports estimating the rates to be closer to 90 to 95%. Suzuki et al. also suggested that variant allele frequency, rather than general mutation positivity, correlated with reduced survival [26]. Their study assessed a multi-institutional cohort of 1162 pancreatic cancer patients with resectable tumors, utilizing multiplex droplet digital polymerase chain reaction (ddPCR) to identify common mutations and copy number alterations in codons 12, 13, and 61 from low-cellularity biopsy samples. The researchers differentiated wt*KRAS* and m*KRAS* by the presence of at least 1% allele variants. Using that threshold, *KRAS* mutations did not significantly correlate with overall survival (OS) or disease-free survival (DFS). Instead, high VAF of m*KRAS* was associated with negative prognostic markers, including reduced OS and DFS, large tumor size, adenosquamous histology, neurovascular invasion, and high tumor cellularity.

We (Nauheim et al. [27]) have also reported on our findings of *KRAS* VAF-dependent prognostic impact in a cohort (*n* = 144) of early stage, resectable PDAC patients. These observations were based on data from a single institution that employed a 10% variant allele frequency reporting threshold (corresponding to a 5% heterozygous mutation frequency). Significant prognostic differences were noted between cases of low (<20%) and high (≥20%) *KRAS* VAF [27]. That study provided supporting evidence for the reduced DFS, larger tumors, and greater tumor cellularity seen in high-VAF cases in other studies. In addition, high VAF was found to correspond with more advanced tumor stages and a higher rate of distant recurrence as compared to patients with low VAF. These studies are summarized in Table 3 below.

Mechanistically, Knudsen et al. [28] have shown that VAF corresponds with tumor heterogeneity, noting that high VAFs were found in key genetic drivers such as *KRAS*, *TP53*, and *SMAD4*, which tended to remain conserved over multiple passages of cell culturing. Similarly, upon comparison between primary tumors and patient-derived xenograft models (PDXs), high-VAF mutations tended to remain stable and not change, yielding a similar heterogeneity of *KRAS* mutant alleles in both primary tumor samples and in subsequent tumors passaged and grown as mouse xenografts. They have also found that since KRAS is the main and common driver of most PDAC, low *KRAS* VAF was associated with higher VAF of other mutational genetic drivers. 

**Table 3:** Reported clinical impact of KRAS variant allele frequencies (VAFs) on survival in pancreatic cancer.

Study	Cohort	Findings
Lennerz et al. [25], Biankin, et al. [29]	Early stage (Stage I–II)	Mutant *KRAS* VAF ≥ 10% was associated with a trend of worse overall survival compared with VAF < 10%
Suzuki et al. [26]	All stages (I–IV)	Both overall and disease-free survival showed a ‘dose-dependent’ impact of mutant *KRAS* VAF, with VAF < 10% showing the best survival and VAF ≥ 20% showing the worst survival.
Nauheim et al. [27]	Early stage (Stage I–II)	Mutant *KRAS* VAF ≥ 20% patients presented with a more aggressive, advanced-stage disease, and were noted to have worse disease-free survival.

## 5. Targeted Therapy

As KRAS mutations and the RAS pathway are recognized as a primary driver of PDAC progression and resistance to therapy, many attempts have been made to target KRAS activity. For many years, KRAS was considered ‘undruggable’ due to three main barriers: (a) its smooth three-dimensional conformation, which lacks any distinct ‘bindable’ pockets, making the design of relevant ligands extremely challenging; (b) RAS proteins are strong binders of GTP at even sub-nanomolar concentrations; and (c) intracellular GTP concentrations are relatively 1000-fold higher than the affinity constant of RAS and GTP binding [30,31].

The differing effects of KRAS variants on clinical outcomes in PDAC patients have prompted drug developers to pursue targeted KRAS inhibitor therapies [31]. Several approaches were used to target KRAS over the years: AZD4785, an anti-KRAS antisense oligonucleotide that had promising pre-clinical results in in vitro and in vivo animal models, failed to show any benefit during its phase I, open-label, multicenter, dose-escalation clinical study in 2019 (NCT03101839). A KRAS G12D siRNA study, based on exosome delivery, is currently enrolling metastatic PDAC patients (NCT03608631) [32]. Other drugs, such as CPD0857, have been used to target KRAS through the promotion of ubiquitin-related degradation, via the Wnt/β-catenin pathway, as in the case of KYA1797K [33,34].

A major breakthrough in targeting KRAS came in 2021, when the Food and Drug Administration (FDA) approved sotorasib (AMG-510) for the treatment of KRAS G12C-specific mutations for advanced non-small cell lung cancer (NSCLC) in patients previously treated with at least one systemic therapy. The approval leveraged the CodeBreaK 100 clinical trial results that demonstrated an overall response rate (ORR) of 36% with a median response duration of 10.0 months (95% CI) [35]. Sotorasib irreversibly binds the GDP-bound form of KRAS, which stabilizes the protein’s inactive conformation, inhibits tumor cell growth, and promotes apoptosis. Strickler et al. [36] investigated the safety and efficacy of sotorasib in pancreatic cancer patients with KRAS G12C mutations, present in 1–2% of pancreatic cancers. Their phase 1 and phase 2 trials of 38 metastatic patients with prior chemotherapy were found to have an ORR of 21% with a median overall survival of 6.9 months (95% CI).

Another G12C inhibitor, adagrasib (MRTX849), with demonstrated activity in KRAS G12C-mutated NSCLC and colorectal cancer (CRC), has recently shown effectiveness in PDAC and other solid tumors (NCT03785249) [37]. Specifically, a 33.3% ORR in 7 out of 21 patients was found in patients with PDAC. The most common adverse effects were nausea, diarrhea, fatigue, and vomiting, found in 49.2%, 47.6%, 41.3%, and 39.7%, respectively, of the general study population. Although adverse effects led to dose reduction or interruption in some patients, there was no discontinuation of adagrasib due to adverse effects. 

Since G12D is the predominant KRAS mutation in PDAC, significant efforts are ongoing to target this isoform. The small-molecule inhibitor MRTX1133, the molecular structure of which is based on MRTX849, has shown promise in mouse models to reversibly inhibit KRAS G12D by preventing guanine nucleotide exchange. Phase 1–2 clinical trials are currently underway to find the optimal dose of MRTX1133 in solid tumor patients with colorectal, pancreaticobiliary, or NSCLC-bearing G12D mutations. In vivo xenograft mouse models have demonstrated highly potent and selective antitumor activity with a dose-dependent response [38]. In a murine model, intraperitoneal administration of MRTX1133 at 3 mg/kg BID produced 94% growth inhibition. Tumor regression was seen at higher doses, 62% with 10 mg/kg BID and 73% with 30 mg/kg BID. These promising results await clinical trial confirmation.

Another interesting inhibitor is the pan-RAS inhibitor, RMC6236, targeting NRAS, HRAS, and KRAS, which is assessed in the NCT05379985 phase I clinical study [39]. Although the study is still ongoing, initial data are encouraging and suggest a possible encouraging response in multiple mutant KRAS subtypes, based on circulating DNA measurement [40]. Early results presented at the 2023 ESMO Congress in Madrid, Spain, October 20–24 [41], showed an objective response rate (ORR) to RMC-6236 in NSCLC of 38%, with a disease control rate (DCR) of 85%. In pancreatic ductal adenocarcinoma patients, early results of RMC-6236 suggested an ORR of 20% with a DCR of 87%, with also a marked reduction in the measurement of mutant KRAS alleles in circulating tumor DNA studies.

Two immune-based approaches include targeted vaccines and T-cell-based adaptive immunotherapy. The NCT03592888 clinical trial is investigating a dendritic cell vaccine combined with patient-specific mutant KRAS peptides tailored to the patients’ specific KRAS mutation subtype and human leukocyte antigen type. This combined vaccine-peptide therapy is administered twice, 8 weeks apart, in resectable pancreatic cancer patients and evaluated for disease recurrence up to 1 year after treatment [42]. Another phase 1 study, NCT04117087, is studying the effect of a pooled long peptide vaccine targeting mutant KRAS co-administered with the immune checkpoint inhibitors nivolumab and ipilimumab in resected PDAC patients post adjuvant or neoadjuvant treatment, and in metastatic mismatch-repair-proficient colorectal cancer patients [43]. An immunotherapy consisting of lipid-conjugated oligonucleotides and peptides targeting G12D and G12R is also being assessed as adjuvant treatment for PDAC patients in the trial NCT04853017 [44]. 

Two currently accruing studies using adaptive immunotherapy include (1) the ongoing NCT03745326 Gilead-sponsored trial, currently enrolling phase I-clinical study, estimated to conclude by 2028, attempting to target G12D mutant KRAS expressing cells by modifying patient T cells with anti-KRAS G12D murine TCR expression [45], and (2) the NCT04146298 trial, estimated to conclude by 2025, which modifies T cells with anti-KRAS G12V TCR [46].

In light of the growing research into precision medicine targeting KRAS mutant subtypes, we need to consider the applicability and future of next-generation sequencing and molecular profiling of pancreatic cancer in the clinical setting. Currently, the National Comprehensive Cancer Network guidelines for pancreatic cancer [47] recommend somatic molecular profiling in cases of unresectable, recurrent, and/or metastatic disease for pre-treatment evaluation. They further recommend testing for specific gene fusions, RAS/RAS driver mutations, homologous repair (BRCA1, BRCA2, PALB2) and mismatch repair mutations (MLH1, MSH2, MSH6, PMS2), as well as tumor microsatellite instability and tumor mutational burden. However, there are no comparable current guidelines for the profiling of resectable, early stage tumors. There are, of course, multiple reasons to support the application of molecular profiling to early stage tumors: Multiple studies have suggested improved adjuvant therapy results when taking tumor molecular profiles into consideration. These include chemotherapy response prediction models, such as GemPred for gemcitabine response [48,49] and SMAD4 alterations for FOLFIRINOX response [50]. Furthermore, as PDAC is associated with a high rate of recurrence and metastasis, even in early stage tumors, prior knowledge of the primary tumor’s molecular profile could prove to be invaluable, especially in cases where the recurrent/metastatic lesion is too small to sample or analyze. This rationale is supported by the fact that metastatic PDAC lesions seem to bear a close similarity to their primary tumors, as reported clinically by Brar et al. [51] and experimentally by Knudsen et al. [28]. Therefore, at our practice, we routinely utilize a next-generation sequencing panel assessing hotspot mutation regions in 42 clinically relevant cancer genes (ABL1, AKT1, ALK, APC, ATM, BRAF, CDH1, CSF1R, CTNNB1, EGFR, ERBB2, ERBB4, FBXW7, FGFR1, FGFR2, FLT3, GNA11, GNAQ, GNAS, HRAS, IDH1, JAK2, JAK3, KDR, KIT, KRAS, MET, MLH1, MPL, NOTCH1, NPM1, NRAS, PDGFRA, PIK3CA, PTEN, PTPN11, RET, SMAD4, SMARCB1, SMO, SRC, and TP53) [52]. This panel is often complemented by MMR and MSI testing as well as BRCA1, BRCA2, and PALB2. As multiple research centers are creating tumor tissue banks, and profiling tumors as part of their clinical research strategy, we expect to see more studies, retrospective and prospective, leveraging early stage profiling to inform adjuvant treatment decisions.

## 6. Conclusions

KRAS mutations play a critical role in PDAC progression and chemotherapy resistance. Different mutation subtypes affect various regions on the KRAS protein and affect binding affinity to other regulatory proteins, which supports the observation of differential oncologic outcomes between the various subgroups. While there is a complex interplay between the different driver oncogenes, tumor suppressor proteins, and their respective subtypes, numerous studies have identified the G12D subtype as carrying the worst prognosis in all cancer stages. Similarly, increased allele frequency of G12D also appears to be a negative predicator of survival. The increased interest in and focus on the specific KRAS mutation subtypes has led to the development of exciting and novel targeted therapies, which are currently being evaluated in clinical studies. It is anticipated that future studies will contribute to further improvement in the survival of PDAC patients and that, in the not too distant future, true “personalized therapy” will become a reality.

## Figures and Tables

**Table 1:** Five-year survival rates of pancreatic cancer patients by stage. Data from the SEER database 2023 [1].

Pancreatic Cancer Stage	Degree of Spread	5-Year Survival Rate (%)
Stage I	Localized	44
Stage II/III	Regional	15
Stage IV	Distant	3

**Table 2:** Reported oncological outcomes in PDAC based on KRAS mutation subtype.

Study	Cohort	Findings
Hendifar et al. [19]	Advanced disease	KRAS G12R and KRAS G12V have greater overall survival than KRAS G12D in advanced-stage PDAC.
Chen et al. [12]	Advanced disease	ctDNA analysis revealed mutant KRAS was associated with shorter OS than patients with wild-type KRAS.
Diehl et al. [18]	Advanced Disease	KRAS G12R was associated with improved OS and PFS compared with non-G12R.
Ogura et al. [13]	Advanced disease	KRAS G12D and G12R mutations had shorter overall survival than G12V, which had shorter survival than wild-type KRAS in unresectable pancreatic cancer patients.
Dai et al. [9]	Resectable Disease	KRAS G12D was associated with worse survival compared with wild-type KRAS.
Kawesha et al. [20]	Resectable Disease	KRAS G12V and KRAS wild-type had longer overall survival than KRAS G12D in patients who had undergone resection for PDAC.
Schultz et al. [24]	Resectable Disease	Mutant KRAS was not associated with RFS and OS compared with wild-type KRAS.
McIntyre et al. [14]	Resectable Disease	Mutant KRAS correlated with worse survival compared with wild-type KRAS.
Shen et al. [21]	Resectable Disease	KRAS G12D in resectable PDAC patients had shorter overall survival than non-G12D patients.
Guo et al. [23]	Resectable disease	ctDNA analysis revealed mutant KRAS detection to be strongly associated with worse OS and RFS. G12D mutations were strongly, correlated with poor OS and RFS.
Bournet et al. [17]	All stages	Mutant KRAS and wild-type KRAS had comparable survival. G12D had significantly worse survival.
Yousef et al. [15]	All stages	KRAS G12D and Q61H mutations had shorter overall survival, while G12R and wild-type KRAS had improved and comparable survival.
Windon et al. [10]	All stages	Mutant KRAS correlated with worse survival compared with wild-type KRAS.
Zhou et al. [16]	All stages	KRAS mutation status did not correlate with survival.
Shoucair et al. [22]	All stages	KRAS G12D co-occurrence with mutant TP53 was associated with improved survival.

## Data Availability

Not applicable.
